# Effect of LXR/RXR agonism on brain and CSF Aβ40 levels in rats

**DOI:** 10.12688/f1000research.7868.2

**Published:** 2016-04-19

**Authors:** Songli Wang, Paul Wen, Stephen Wood

**Affiliations:** 1 Genome Analysis Unit, Amgen Inc., San Francisco, CA, USA; 2 Department of Neuroscience, Amgen Inc., Thousand Oaks, CA, USA

**Keywords:** Alzheimer’s, Apolipoprotein E, Aβ, liver X receptor, retinoid X receptor, Bexarotene

## Abstract

Alzheimer's disease (AD) is characterized pathologically by the presence of amyloid plaques and neurofibrillary tangles. The amyloid hypothesis contends that the abnormal accumulation of Aβ, the principal component of amyloid plaques, plays an essential role in initiating the disease. Impaired clearance of soluble Aβ from the brain, a process facilitated by apolipoprotein E (APOE), is believed to be a contributing factor in plaque formation. APOE expression is transcriptionally regulated through the action of a family of nuclear receptors including the peroxisome proliferator-activated receptor gamma and liver X receptors (LXRs) in coordination with retinoid X receptors (RXRs). It has been previously reported that various agonists of this receptor family can influence brain Aβ levels in rodents. In this study we investigated the effects of LXR/RXR agonism on brain and cerebrospinal fluid (CSF) levels of Aβ40 in naïve rats. Treatment of rats for 3 days or 7 days with the LXR agonist, T0901317 or the RXR agonist, bexarotene did not result in significant changes in brain or CSF Aβ40 levels.

## Introduction

Alzheimer’s disease (AD) is a debilitating neurodegenerative disease and the leading cause of dementia in the elderly. It is currently estimated that 5 million people in the US and 30 million worldwide are afflicted with this disease. The pathological hallmarks of AD are the presence of extracellular amyloid plaques and intracellular neurofibrillary tangles in the hippocampus and cortical areas of the brain ^1^. The core constituent of the amyloid plaques is a 4 kDa peptide known as amyloid-β peptide (Aβ). Aggregation of Aβ into soluble, multimeric assemblies and insoluble amyloid fibrils is hypothesized to contribute directly to the pathogenesis of AD; therefore therapeutic strategies aimed at lowering soluble Aβ levels in the brain would be predicted to have a disease-modifying effect ^2^.

The E4 allele of apolipoprotein E (APOE) is the largest genetic risk factor for sporadic, late-onset AD. The presence of a single copy of E4 increases the risk for Alzheimer’s disease 3-fold and individuals with 2 copies are 15 times more likely to develop AD ^3^. Data showing that APOE4 carriers begin to accumulate amyloid deposits earlier in life relative to non-carriers ^4^ has led to the hypothesis that increased risk associated with an E4 genotype may be the result of the effects of APOE on Aβ production, turnover and/or clearance from the central nervous system (CNS).

The expression of genes encoding lipid-transport proteins, including APOE is transcriptionally regulated by the ligand-activated nuclear receptors, peroxisome proliferator-activated receptor gamma (PPARγ) and liver X receptors (LXRs) which form obligate heterodimers with retinoid X receptors (RXRs) ^5^. Additionally, activation of these receptors has been shown to affect the activation state of macrophage and microglia ^6^. Based on the processes influenced by this nuclear receptor family it is a reasonable hypothesis that agonism of one or more members of the family could have beneficial effects on Aβ homeostasis in the CNS. In fact, several groups have demonstrated that LXR agonism with either GW3965 ^7^ or T0901317 ^8– 11^ results in reduced amyloid plaque burden and/or soluble Aβ levels in amyloid precursor protein (APP) transgenic mouse models. Using non-transgenic rats, Suon *et al.* demonstrated a statistically significant increase in CSF Aβ and a decrease in soluble brain Aβ following T0901317 treatment ^12^. In addition, it was reported that a highly selective, blood-brain-barrier–permeant, RXR agonist, bexarotene (Targretin), enhanced clearance of soluble Aβ in an APP transgenic mouse model in an APOE-dependent manner. In the same study, Aβ plaque burden was reduced by more than 50% within 72 hours. Further, bexarotene treatment also resulted in a similar reduction (~25%) in brain interstitial fluid (ISF) levels of Aβ in non-transgenic, C57Bl/6 mice 7–12 hours following a single administration ^13^. Attempts to replicate the bexarotene findings resulted in mixed results (see reviews by Tesseur & De Strooper ^14^ and Tousi ^15^). This study aims to examine the robustness of the hypothesis that RXR or LXR agonism affects soluble Aβ homeostasis in the CNS.

## Materials and methods

***In vivo* pharmacodynamic studies:** All procedures were approved by the Amgen Institutional Animal Care and Use Committee. Young male Sprague-Dawley rats (175–200 g) were purchased from Harlan (Indianapolis, IN) and were maintained on a 12h light/dark cycle with unrestricted access to food and water until use. Rats were dosed orally for 3 and 7 consecutive days with AMG8155, a proprietary small molecule BACE1 inhibitor, at 3 mg/kg in 2% HPMC and 1% Tween 80, pH 2, bexarotene (Alfa Aesar, Ward Hill, MA) at 100 mg/kg in 30% Labrasol, 1% Tween 20, 2% Providone and 0.05% BHA, pH7.0 (Vehicle 3), and T0901317, a LXR agonist (Fisher Scientific, Pittsburgh, PA), at 30 mg/kg in 0.5% NaCl, 2% Tween 80 (Vehicle 4). 4 hours post dose on the last day of study, rats were euthanized with CO _2_ inhalation for 2 minutes and the cisterna magna was quickly exposed by removing the skin and muscle above it. Cerebrospinal fluid (CSF) was collected with a 30 gauge needle inserted through the dura membrane covering the cisterna magna. CSF samples with visible blood contamination were discarded. Blood was withdrawn by cardiac puncture and plasma was obtained by centrifugation at 15,000 rpm for 10 min at 4°C for drug exposure. Brains were removed and, along with the CSF, immediately frozen on dry ice and stored at -80°C until use. The frozen brains were subsequently homogenized in 10 volumes (w/v) of 0.5% Triton X-100 in TBS with protease inhibitors cocktails. The homogenates were centrifuged at 355,000 rpm for 30 min at 4°C.

**Quantification of Aβ40 and APOE in brain and CSF**: Samples are analyzed for Aβ levels by immunoassay with a MSD imager. Briefly, 96-well avidin plates (MesoScale Discovery, Inc., Gaithersburg, MD) were coated with biotinylated-anti-Aβ antibody 4G8 (mouse monoclonal, Cat# Sig 39240-1000, Covance Research Products, Princeton, NJ) at 10 μg/ml in PBS. Samples were co-incubated in the plate overnight at 4°C along with a ruthenium-labeled anti-Aβ antibody specific for the C-terminal region of Aβ40 (ConFab40; Amgen, Thousand Oaks, CA). Plates were then washed, 150 μl/well read buffer T (MesoScale Discovery, Inc.) was added, and plates were read immediately on a Sector 6000 imager according to the manufacturer’s recommended protocol (MesoScale Discovery, Inc.). All samples were assayed in triplicate and analyzed by using Prism version 5.04 (GraphPad Software Inc., San Diego, CA). Data was analyzed by one-way analysis of variance and Dunnett’s multiple comparison test.

APOE levels in brain (50 μg homogenates) and CSF (10 μl) were analyzed by Western blot following PAGE using 4–12% Bis-Tris gels (Invitrogen, Carlsbad, CA). Blots were probed with primary antibodies to APOE (goat polyclonal, EMD Millipore; 1:1000) and the loading control, actin (ThermoFisher Scientific; 1:200) for 60 min at 4°C and then washed with TBST (Tris-buffered saline, 0.1% Tween 20) three times at room temperature, followed by (Goat-anti-mouse) secondary antibody (ThermoFisher Scientific; 1:1000) for 30 min at 4°C. Densitometric analysis of ApoE was performed (exposure time of 4 minutes with a relative intensity of 2.0, Odyssey imaging system, with application software Version 3.0) followed by an unpaired t-test using GraphPad Prism 5.04 software.

**Measurement of Plasma, CSF, and Brain Drug Concentration:** Aliquots of plasma (50 μl) were combined with 300 μl of acetonitrile containing 125 μl structurally related internal standard (IS), vortexed, and centrifuged. Supernatant was transferred into a plain polypropylene 96-well plate for sample analysis. Brain tissue samples were homogenized by using a Covaris (Woburn, MA) acoustic homogenizer. Aliquots of 50 μl homogenate were combined with acetonitrile containing a structurally related IS, vortexed, and centrifuged at 1,900 g for 5 minutes. Supernatant was transferred into a 96-well plate for sample analysis. Analytical standards and tissues were measured by liquid chromatography mass spectrometry (Shimadzu Pumps Autosampler Prominence for HPLC and PE Sciex API 4000 for MS, with Analyst 1.6.1 software) using atmospheric-pressure chemical ionization and multiple reaction monitoring in the positive ion mode.

## Results

Our aim in this study was to investigate the effects of RXR/LXR agonism on Aβ homeostasis in the CNS of non-transgenic rats using the RXR agonist, bexarotene and the LXR agonist, T0901317. As a positive control, we included a β-secretase inhibitor (AMG8155). Compounds and appropriate vehicle controls were administered to naïve Sprague Dawley rats at doses indicated in Table 1 for either 3 or 7 consecutive days.

**Table 1.** Dosing Table.

Group	Dose (mg/kg)
Bexarotene	100
AMG8155	3
T0901317	30

Table 1 lists the 3 compounds tested in this study along with the respective doses (mg/kg).

**Figure 1.**
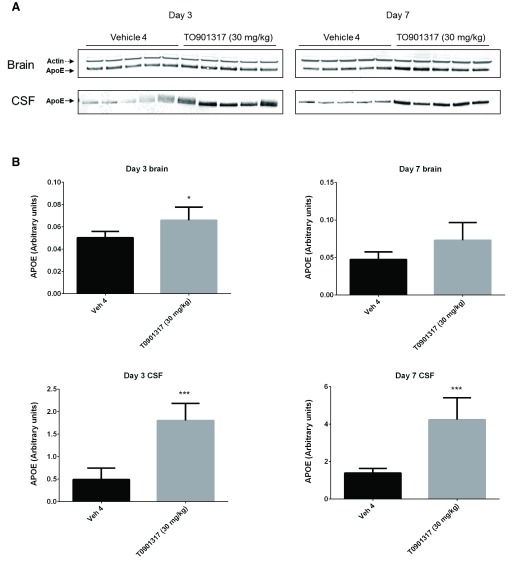
LXR agonist, T0901317 significantly increased APOE levels in rat CSF following 3 and 7 days of dosing at 30 mg/kg. APOE was also increased in brain however the changes only reached statistical significance at day 3. **A**) Western blot analysis of APOE in brain and CSF. **B**) Densitometric analysis of the bands was performed as described in the Materials and Methods section; data are presented as the mean plus standard deviation; Vehicle 4 (black bars) and T0901317 (gray bars).

**Figure 2.**
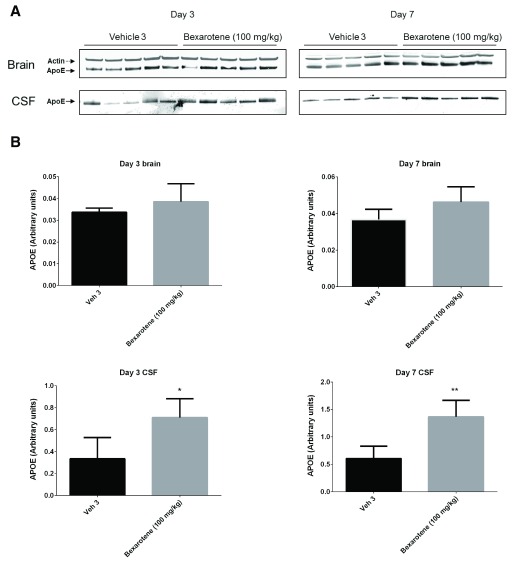
RXR agonist, bexarotene significantly increased APOE in rat CSF following 3 and 7 days of dosing at 100 mg/kg. APOE changes in brain were not statistically significant. **A**) Western blot analysis of APOE in brain and CSF. **B**) Densitometric analysis of the bands was performed as described in the Materials and Methods section; data are presented as the mean plus standard deviation; Vehicle 4 (black bars) and bexarotene (gray bars).

**Figure 3.**
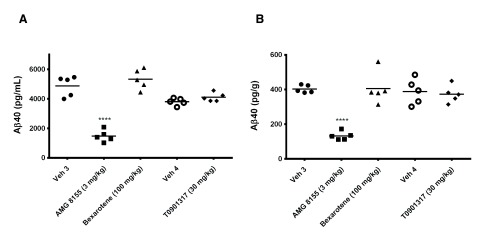
Aβ40 levels in ( **A**) CSF and ( **B**) brain were unchanged following 3 days of treatment with bexarotene (triangles) or T0901317 (diamonds). Positive control BACE inhibitor AMG8155 (squares) reduced Aβ40 levels 70 and 67% in CSF and brain respectively following a single administration.

**Figure 4.**
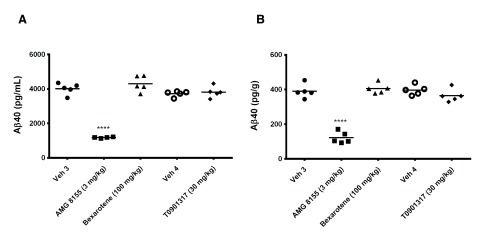
Aβ40 levels in ( **A**) CSF and ( **B**) brain were unchanged following 7 days of treatment with bexarotene (triangles) or T0901317 (diamonds). Positive control BACE inhibitor AMG8155 (squares) reduced Aβ40 levels 71 and 69% in CSF and brain respectively following a single administration.

Following 3 and 7 days of dosing, animals were evaluated for both compound levels and pharmacodynamic endpoints. APOE levels were quantitated in brain homogenate and CSF by Western blot. Aβ40 levels were quantitated in the same compartments using immunoassay as described in the Materials and methods section. Following 3 and 7 days of dosing, APOE levels were increased in brain and CSF in the T0901317 treated animals compared to vehicle treated animals ( Figure 1). Changes in CSF were statistically significant at both 3 (p = 0.0002) and 7 days (p = 0,0007) whereas changes in brain were statistically significant at day 3 (p = 0.030) but did not reach significance at day 7 (p = 0.056). Bexarotene treatment also resulted in a statistically significant increase in CSF APOE levels compared to vehicle treated animals following both 3 (p = 0.019) and 7 days (p = 0.002) of dosing ( Figure 2). APOE levels in brain following bexarotene treatment trended towards an increase however these changes were not statistically significant. Soluble Aβ40 levels were unchanged in brain and CSF following 3-day ( Figure 3) and 7-day ( Figure 4) treatment with either bexarotene or T0901317. The positive control BACE inhibitor, AMG8155 effectively reduced Aβ40 levels by 70% and 71% in CSF and by 67% and 69% in brain in the 3-day and 7-day studies respectively ( Figure 3 and Figure 4).

Drug levels of bexarotene and T0901317 were measured in plasma and brain homogenate following 3 and 7 days of dosing ( Table 2). Total levels of both compounds achieved single-digit to low double-digit μM levels in brain and showed good uptake in brain relative to plasma in both dosing paradigms.

**Table 2.** Compound Exposure Table.

Treatment Duration	Compound	[brain] _t_, μM	[plasma] _t_, μM	[brain] _t_/ [plasma] _t_
3 days	Bexarotene (100 mg/kg)	6.26	5.74	1.09
	T0901317 (30 mg/kg)	14.44	5.30	2.72
7 days	Bexarotene (100 mg/kg)	3.41	4.76	0.72
	T0901317 (30 mg/kg)	11.68	4.30	2.72

Following 3 and 7 days of dosing, compound levels were measure in brain homogenate and plasma. Total (t) compound concentrations (μM) are reported in each case. The brain to plasma ratio is also shown (far right-hand column).

## Conclusion

In this study we demonstrate that 3-day or 7-day treatment of naïve rats with the LXR agonist, T0901317 or the RXR agonist, bexarotene treatment results in an increase in APOE levels in CSF without observable effects on CSF or brain Aβ40 levels.

Although this study sought to examine the robustness of the hypothesis that RXR/LXR agonism affects soluble Aβ homeostasis, it was not designed to explicitly replicate any one prior study. Differences in findings between labs could very well be a result of a variety of factors related to the methodologies employed, something that has been nicely reviewed for bexarotene previously ^14, 15^.

Whereas the current report was focused solely on soluble Aβ, we recognize that RXR/LXR agonism may affect AD pathology in other ways (e.g. increased phagocytic clearance of amyloid deposits ^16^). LXR or RXR agonism may also affect cognitive decline in AD patients via non-Aβ-dependent mechanisms not yet fully understood ^17^. Moreover, this study exclusively assessed Aβ40 and it remains possible that LXR/RXR agonism may result in Aβ42-specific changes. In most prior studies that have examined changes in soluble Aβ40 and Aβ42 homeostasis, both Aβ species are affected in a similar manner. However, one published report showed that LXR agonism with T0901317 resulted in a selective reduction in Aβ42 in the hippocampus only ^11^. Such region-specific changes would not have been detected under our current experimental protocol as whole brain homogenates were analyzed.

Finally, others have shown that the effects of LXR/RXR agonism vary depending on APOE isoform. Treatment of EFAD mice (mice expressing 5XFAD mutations and h-APOE3 or h-APOE4) ^18^ with bexarotene or bexarotene analog, LG100268, resulted in an increase in APOE4 lipidation and subsequent decrease in soluble, oligomeric Aβ levels ^19^. Likewise, in naïve human APOE3 or APOE4 targeted replacement mice, bexarotene treatment increased APOE4 lipidation and decreased E4-associated Aβ42 and hyperphosphorylated tau accumulation in the hippocampus ^20^. The current study was performed in naïve rats expressing endogenous APOE, therefore, human APOE isoform-specific effects would have been beyond the scope of this study.

It remains to be seen how well any of these preclinical findings translate to human clinical trials. Recently, the effect of bexarotene on amyloid burden was assessed in patients with Alzheimer's disease (AD) in a small, proof-of-concept trial ^21^. Although the primary outcome of the trial was negative, data suggest that bexarotene resulted in lowering of amyloid burden in APOE4 non-carriers.

We hope that these findings will stimulate future discussion in the Alzheimer’s research community on the impact of LXR/RXR agonism on central Aβ homeostasis.

## Data availability

The data referenced by this article are under copyright with the following copyright statement: Copyright: © 2016 Wang S et al.

Open Science Framework: Dataset: Effect of LXR/RXR agonism on brain and CSF Aβ40 levels in rats, doi: 10.17605/OSF.IO/3NS64 ^22^
