# Supplementary material for: Effect of LXR/RXR agonism on brain and CSF Aβ40 levels in rats
Source: F1000Res. 2016 Apr 19;5:138. Originally published 2016 Feb 4. [Version 2] doi: 10.12688/f1000research.7868.2 (PMC4866632; doi:10.12688/f1000research.7868.2)
Supplement: Peer review history

# Peer review: Referee response for version 2

**DOI:** 10.5256/f1000research.9090.r12557

**Published:** 2016-05-11

**Authors:** Daniel M. Michaelson

**Affiliations:** 1 Department of Neurobiology, Sagol School of Neurosciences, Myriam Lebach Chair in Molecular Neurodegeneration, The George S. Wise Faculty of Life Sciences, Tel Aviv University, Tel Aviv, Israel

This communication revealed that treatment of rats with the  LXR/RXR  agonists bexarotene and T0901317 increased the brain levels of apoE but had no effect on the levels of Ab40.

A key issue which is not yet resolved in this paper, and which I believe is important, is whether the present treatments with the LXR/RXR agonists in the rat result in partial/weak activation of the LXR/RXR system which  affects the levels of apoE but not of the other related readout parameter ( i.e. Ab40 in this case) or that the LXR/RXR system  is robustly activated by the presently employed paradigm  but that this does not affect the levels of Ab40.

Assessment of the "on target" effects of such pharmacological manipulations is important for determination of the significance and meaning of the Ab40 related negative finding and for addressing and reconciling difference between this study and previous reports (eg ref 12 of Suon et al 2010). Accordingly, we suggest that the expression of  additional LXR\RXR driven genes be monitored ( eg ABCA1 by either western blots or PCR)  and if possible also Ab42.

The Suon study was performed utilizing a forebrain + hippocampus brain extract of 1 month old rat. The possible role of differences in these parameters in mediating the differences between the effects of the RXR\RXR  treatment in the Suon study and the present manuscript should be discussed.

I have read this submission. I believe that I have an appropriate level of expertise to confirm that it is of an acceptable scientific standard, however I have significant reservations, as outlined above.

# Peer review: Referee response for version 1

**DOI:** 10.5256/f1000research.8469.r12696

**Published:** 2016-03-02

**Authors:** Mary Jo LaDu, Conor Smith, Ana Valencia

**Affiliations:** 1 Department of Anatomy and Cell Biology, University of Illinois at Chicago, Chicago, IL, USA

In this manuscript, Wang and colleagues report that treatment for 3 and 7 d bexarotene (Bex, an RXR agonist) or T0901317 (TO, an LXR agonist) induce an increase in apoE levels and but have no effect on Aβ40 in the CSF and brain of rats. While the subject matter is timely, the conclusions must be taken with reservations because of three major concerns:

 

1. Limited readouts and regions analyzed. It has been previously demonstrated that changes in the levels of Aβ40 and apoE in CSF and brain do not always correlate with efficacy in FAD-Tg mice ^1^^,^^2^. Furthermore, it has been shown that an increase of apoE levels by Bex can be beneficial or detrimental depending on the isoform of apoE ^3^ or the brain region analyzed ^3^.
2. The exclusion of Aβ42. It has been reported that CSF Aβ42 levels, and not Aβ40, are increased in AD patients compared to controls ^4^.
3. Comparison to previous reports. Suon and colleagues (2010) ^5^ treated rats with LXR agonists TO (at the same dose) or GW3965 and reported an increase in apoE that correlated with an increase in Ab40/42 levels in CSF and a reduction of Aβ40 in brain ^5^. Wang and colleagues should reconcile their results with this work specifically and interpret their work in the context of the filed in general.

A minor observation is that in Figure 1A the vehicle is mislabeled as Vehicle 3 (should be Vehicle 4 for TO901317)

Within the AD field, there is an ongoing discussion of the effects of Bex on soluble and deposited Aβ levels in the brain and cognition, with contradictory findings. This is likely due to several confounding factors, including: variance within and across models and inconsistencies in the methods used to characterize and quantify the proteins of interest, particularly soluble Aβ. It is imperative to take these parameters into account when considering the efficacy of RXR or LXR agonists for AD therapeutics. As well, a phase I trial of Bex in AD patients recently concluded ^6^. While these results may be of limited significance and were not available for the submission of this manuscript, inclusion of their results in a revision of the Discussion would be helpful.

We have read this submission. We believe that we have an appropriate level of expertise to confirm that it is of an acceptable scientific standard, however we have significant reservations, as outlined above.

## References

[1] TesseurILoACRoberfroidADietvorstSVan BroeckBBorgersMGijsenHMoecharsDMerckenMKempJD'HoogeRDe StrooperB: Comment on. *Science*.2013;340(6135) : 10.1126/science.1233937 924-e 10.1126/science.1233937 23704554

[2] Boehm-CaganAMichaelsonDM: Reversal of apoE4-driven brain pathology and behavioral deficits by bexarotene. *J Neurosci*.2014;34(21) : 10.1523/JNEUROSCI.5198-13.2014 7293-301 10.1523/JNEUROSCI.5198-13.2014 24849361PMC6608187

[3] TaiLMKosterKPLuoJLeeSHWangYTCollinsNCBen AissaMThatcherGRLaDuMJ: Amyloid-β pathology and APOE genotype modulate retinoid X receptor agonist activity in vivo. *J Biol Chem*.2014;289(44) : 10.1074/jbc.M114.600833 30538-55 10.1074/jbc.M114.600833 25217640PMC4215234

[4] BlennowKMattssonNSchöllMHanssonOZetterbergH: Amyloid biomarkers in Alzheimer's disease. *Trends Pharmacol Sci*.2015;36(5) : 10.1016/j.tips.2015.03.002 297-309 10.1016/j.tips.2015.03.002 25840462

[5] SuonSZhaoJVillarrealSAAnumulaNLiuMCarangiaLMRengerJJZerbinattiCV: Systemic treatment with liver X receptor agonists raises apolipoprotein E, cholesterol, and amyloid-β peptides in the cerebral spinal fluid of rats. *Mol Neurodegener*.2010;5: 10.1186/1750-1326-5-44 44 10.1186/1750-1326-5-44 21034469PMC2988784

[6] CummingsJLZhongKKinneyJWHeaneyCMoll-TudlaJJoshiAPontecorvoMDevousMTangABenaJ: Double-blind, placebo-controlled, proof-of-concept trial of bexarotene Xin moderate Alzheimer's disease. *Alzheimers Res Ther*.2016;8(1) : 10.1186/s13195-016-0173-2 4 10.1186/s13195-016-0173-2 26822146PMC4731943

# Peer review: Referee response for version 1

**DOI:** 10.5256/f1000research.8469.r12274

**Published:** 2016-02-29

**Authors:** Sam Gandy

**Affiliations:** 1 Department of Neurology and Psychiatry, Mount Sinai School of Medicine, New York, NY, USA

Some controversy has surrounded the report in *Science* from Landreth and colleagues showing that bexarotene reduces the brain amyloid plaque burden from APP transgenic mice by 50% or more in a matter of days.  Several groups of experts attempted to replicate the dramatic Landreth results but the dramatic results were not replicatable. The current *F1000Research *paper extends that replication attempt story by treating rats with bexarotene and then measuring APP metabolites and apoE in CSF.  In agreement with the "second wave" of bexarotene studies, there was no effect of bexarotene on CSF levels of Aβ40 or Aβ42.  The one point of agreement of all studies was that bexarotene does indeed modulation CSF levels of apoE.  This argues against the development of bexarotene mimetics as Aβ lowering agents for the treatment or prevention of Alzheimer's disease. However, inasmuch as elevating apoE may be beneficial in clinical situations via a non-Aβ-dependent pathway (see http://www.alzforum.org/news/research-news/bexarotene-revisited-improves-mouse-memory-no-effect-plaques), bexarotene does reproducibly modulate CSF levels of apoE.  The discovery of the entity of SNAP (for review, see Jack *et al., *2016 ^1^) indicates that about one-third of clinically diagnosed Alzheimer's patients undergo cognitive decline via some as yet unknown non-Aβ-dependent pathway.

I have read this submission. I believe that I have an appropriate level of expertise to confirm that it is of an acceptable scientific standard.

## References

[1] JackCRKnopmanDSChételatGDicksonDFaganAMFrisoniGBJagustWMorminoECPetersenRCSperlingRAvan der FlierWMVillemagneVLVisserPJVosSJ: Suspected non-Alzheimer disease pathophysiology - concept and controversy. *Nat Rev Neurol*.2016;12(2) : 10.1038/nrneurol.2015.251 117-24 10.1038/nrneurol.2015.251 26782335PMC4784257
